# Case of Rupture of the Uterus—Recovery

**Published:** 1847-08

**Authors:** John S. Davis

**Affiliations:** Dayton, Indiana


ARTICLE VI.

*Case of Rupture of the Uterus—Recovery.* By Dr. John S. Davis, of Dayton, Indiana.

On Monday morning, December 28th, 1846, I was summoned to attend a parturient female, of robust constitution, who had had premonitory symptoms of labor (her sixth pregnancy), some three days in charge of another practitioner. The pains were slight and irregular until Sunday evening, when they suddenly became regular, strong, and expulsive; os uteri dilated; vertix presented; waters drained off and pains continue to increase for some two hours when they suddenly ceased, as the patient expressed "by flying all over her." I found the patient, at 6 o'clock, A. M., free from pain, some nausea, pulse accelerated, weak, and small, uterine globe absent, abdominal parieties flaccid, and abdomen somewhat tender on pressure. On examination per vaginam, in a recumbent posture, I could not reach, with the index finger, the presenting part of the child. Accordingly I had my patient raised to her feet, when I felt the head of the child through the uterine parietes, resting upon the pubis (I found, after delivery, quite a depression in the left parietal bone where it had lodged upon the pubis). The examination was imperfect, as the patient could remain on her feet but a short time, as she became faint and sick. I expressed my fears of the nature of the case to the attending physician, and he thought it impossible, and we gave her the usual doses of ergot, which brought on slight uterine contraction, supposing the difficulty might depend upon atony of the uterus. I now directed the patient to be laid with the perineum free of the edge of the bed, and introduced the hand with the view of bringing down the feet and delivering. I passed the index finger into what I supposed was the os uteri, soft and flaccid, immediately above the symphasis. when my little finger caught a loop of the cord in the hollow of the sacrum. I now directed my attention to this anomoly, when, to my astonishment, I found the os uteri high up in the hollow of the sacrum, with a loop of umbilical cord passed through it. The nature of the case now presented itself. The head of the child had passed into the cavity of the abdomen, through a rupture of the anterior wall of the uterus reaching that portion which looks into the vagina, whilst the body remained within the uterus. I continued the examination, till I learned the os uteri was sufficiently dilatable to admit the hand when I withdrew it and informed the attending physician and friends of the patient of her alarming condition. What was now to be done? We could scarcely hope to be able to return the head within the uterine cavity. However, we resolved to make an effort as no other alternative was left but the Cesarian operation. I accordingly dilated gently the os uteri and passed my hand within and firmly grasped the neck of the child with my fingers against one shoulder and thumb the other, (the palm of the hand looking to the face of the child) and by pushing somewhat forcibly upward and backward, I succeeded in replacing the head within the uterine cavity. I now placed the head in the right iliac fossa, searched for and brought down the feet and soon delivered my patient of a still born child. The uterus contracted pretty firmly upon the placenta and membranes by the assiduous application of gentle friction by the attending physician, and were expelled in due time by the uterine contractions alone. We now placed our patient carefully in bed at 11 o'clock, A. M., gave an opiate and ordered a dose of sulphate of magnesia to be followed in a few hours with another, if the bowels were not moved, and returned home with directions to be sent for should peritonitis supervene before our next visit in the morning. When we arrived at eight o'clock we found our patient in high excitement, which had come on at daylight, with full bounding pulse, hard and frequent, face intensely flushed, and abdomen tender and much swollen, bowels freely moved and unable to urinate. Her arm was tied and blood permitted to flow till a decided impression was made upon the circulation. Urine drawn off with the catheter, and tartarized antimony as largely given as the stomach would bear. She was freely bled again in the evening till the pulse was brought down. Urine drawn off, and antimony continued through the night.

At eight o'clock on Wednesday morning, the condition of our patient was becoming worse—pulse more frequent (one hundred and twenty beats in a minute) and reduced in volume; abdomen more distended and soreness increased; face still flushed and bowels freely moved. We now gave—

℞.—Calomel, grs. xx;
Opii, grs. iii, to be repeated in eight hours—applied evaporating lotions to the abdomen, drew off the urine, and returned home.

At our evening visit we found the patient still worse—pulse in frequency, abdomen more swollen, and soreness so much increased that she could not bear the weight of the hand upon the abdomen. The bladder was again emptied and medicines continued.

On Thursday morning the symptoms were becoming more alarming, pulse more frequent and intermittent at times and smaller in volume, and the tongue which had presented nothing unusual had now became very dry. In the evening copious bilious discharges from the bowels supervened. Calomel discontinued and opium given alone. Urine again drawn off.

On Friday morning the bowels still moved fieely, the discharges serous and frequent; pulse frequent, intermittent, and feeble; abdominal swelling and sourness subsiding.

Things continued pretty much in this condition till Monday morning—abdominal swelling and soreness subsiding; the pulse at times more frequent, feeble, and intermittent; the bladder unable to evacuate its contents, and serous discharge still from the bowels—when the symptoms seemed to improve; pulse became slower and fuller, and not intermittent; aodominal swelling and soreness rapidly subsiding; bladder resumed its functions, and tongue more moist and face less flushed. Our patient continued gradually to improve for some days when she was attacked with phlegmasia dolens in one limb; it was a slight attack and readily yielded to a mercurial purge, followed by aperients and fomentations to the affected limb. It is proper here to observe that the breasts secreted no milk. After the subsidence of the phlegmasia dolens, metritis supervened, attended with much swelling and soreness, a purulent discharge from the vagina, constipated bowels, and slight general fever with white furred tongue. By the use of purgatives and aperients, epispastics and tepid injections into the vagina, together with a gentle mercurial impression kept steadily up for some three weeks, the patient was again convalescent and was able, at the end of two months from her confinement, to walk about the house.

